# Endophytic infection increases the belowground over-yielding effects of the host grass community mainly by increasing the complementary effects

**DOI:** 10.3389/fpls.2023.1191904

**Published:** 2023-06-15

**Authors:** Yaobing Qu, Tianzi Qin, Junzhen Zhang, Yongkang Deng, Xinhe Yu, Xianqin Wei, Nianxi Zhao, Yubao Gao, Anzhi Ren

**Affiliations:** Department of Plant Biology and Ecology, College of Life Sciences, Nankai University, Tianjin, China

**Keywords:** endophyte, *Achnatherum sibiricum*, over-yielding, complementary effects, soil microorganisms

## Abstract

**Introduction:**

Increases in plant species diversity may increase the community diversity effect and produce community over-yielding. Epichloë endophytes, as symbiotic microorganisms, are also capable of regulating plant communities, but their effects on community diversity effects are often overlooked.

**Methods:**

In this experiment, we investigated the effects of endophytes on the diversity effects of host plant community biomass by constructing artificial communities with 1-species monocultures and 2- and 4-species mixtures of endophyte-infected (E+) and endophyte-free (E-) Achnatherum sibiricum and three common plants in its native habitat, which were potted in live and sterilized soil.

**Results and discussion:**

The results showed that endophyte infection significantly increased the belowground biomass and abundance of Cleistogenes squarrosa, marginally significantly increased the abundance of Stipa grandis and significantly increased the community diversity (evenness) of the 4-species mixtures. Endophyte infection also significantly increased the over-yielding effects on belowground biomass of the 4-species mixtures in the live soil, and the increase in diversity effects on belowground biomass was mainly due to the endophyte significantly increasing the complementary effects on belowground biomass. The effects of soil microorganisms on the diversity effects on belowground biomass of the 4-species mixtures were mainly derived from their influences on the complementary effects. The effects of endophytes and soil microorganisms on the diversity effects on belowground biomass of the 4-species communities were independent, and both contributed similarly to the complementary effects on belowground biomass. The finding that endophyte infection promotes belowground over-yielding in live soil at higher levels of species diversity suggests that endophytes may be one of the factors contributing to the positive relationship between species diversity and productivity and explains the stable co-existence of endophyte-infected Achnatherum sibiricum with a variety of plants in the Inner Mongolian grasslands.

## 1 Introduction

Biodiversity has a significant effect on ecosystem functioning, and although no general conclusion has been reached, most studies on the relationship between species diversity and productivity in plant communities have confirmed a positive effect of species diversity on productivity at the local scale (Hector et al., 1999; Tilman et al., 2001; Fridley, 2003), whereas U-shaped, negative, and non-significant effects are also observed (Mittelbach et al., 2001; Gillman and Wright, 2006; Adler et al., 2011; Šímová et al., 2013). The phenomenon that the species in a mixture on average produce more biomass than their respective monocultures is known as over-yielding or net diversity effect (Loreau, 1998; Loreau and Hector, 2001), which is usually explained by complementarity effects and/or selection effects. The complementarity effect occurs because of complementary resource use related to niche partitioning or facilitation among interacting species (Tilman et al., 1997; Loreau, 2000). Selection effects involve an increased probability of more diverse species mixtures harbouring high-yielding species that increase productivity and simultaneously dominate the mixture (Huston, 1997; Tilman et al., 1997). Since Loreau and Hector (2001) presented an approach to separate the complementarity and selection effects on the basis of an additive partitioning analogous to the Price equation in evolutionary genetics, this method has been widely used in experiments investigating the relationship between plant diversity and productivity (Zuppinger-Dingley et al., 2014; Mahaut et al., 2019; Yang et al., 2019; Yi et al., 2021).

Soil microorganisms are considered to be important in regulating plant diversity–productivity relationships (van der Heijden et al., 2008; Hodge and Fitter, 2013). It has been reported that pathogens and mycorrhizal fungi can drive a positive relationship between plant community diversity and productivity (Maron et al., 2011; Schnitzer et al., 2011; Wang et al., 2019). Pathogens are most species-specific in plant monocultures. As plant diversity increases, the species-specific pathogen contents in the soil may be diluted or suppressed, thus promoting a positive diversity–productivity relationship (Maron et al., 2011; Hendriks et al., 2013; Mommer et al., 2018). In contrast, mycorrhizal fungi appear to be less species-specific and can be symbiotic with different plant species in the community. Common mycorrhizal networks may allow the delivery of limiting nutrients, which may favor less competitive plants and contribute to plant co-existence and community diversity effects (Walder et al., 2012). In the soil system, beneficial and pathogenic soil microbes coexist, and their interactive effects on plant diversity–productivity relationships may be further affected by other environmental context (Luo et al., 2017), thus the net effects of soil biota on plant diversity–productivity relationships are complex.

Microorganisms that affect plant growth are diverse and include not only soil microorganisms but also aboveground symbiotic microorganisms such as endophytes. Endophytes are ubiquitous fungi that inhabit the aboveground tissues of healthy plants without causing disease, and *Epichloë* spp. are endophytic clavicipitaceous fungi that form a symbiotic relationship with many cool-season grasses (Arnold et al., 2000). Symbiosis between graminoids and *Epichloë* endophytes occurs in agricultural and natural grassland communities and is often considered reciprocal (Clay, 1990; Clay and Schardl, 2002). In this symbiosis, the host grasses provide shelter, photosynthates and nutrients to the endophytes (Vázquez-de-Aldana et al., 2013). In turn, endophytes benefit grasses through increased growth (Clay, 1990; Saikkonen et al., 2004) and by providing tolerance to abiotic and biotic stresses, including drought (Worchel et al., 2013; Liu et al., 2017), nutrient deficiency (Malinowski and Belesky, 2000; Cheplick and Faeth, 2009), herbivory (Burns and Fisher, 2006) and plant pathogens (Clay and Schardl, 2002; Wang et al., 2016).

*Epichloë* endophytes are able to enhance host plant fitness and interspecific competitive ability (Saikkonen et al., 2013; Vázquez-de-Aldana et al., 2013; Zhou et al., 2018); thus, *Epichloë* endophytes, as symbiotic microorganisms, are also capable of regulating plant communities, but there are few relevant studies on the effects of endophytes on host plant communities. Clay and Holah (1999) found that endophyte infection enhanced tall fescue host dominance and reduced community species diversity but did not affect total community productivity. Afkhami and Strauss (2016) studied the natural grass *Bromus laevipes* and found that endophytes significantly increased host community diversity while increasing host plant dominance but did not affect total community productivity. *Epichloë* endophytes may not only affect the growth and resistances of host grasses, but may also affect soil microbes (Larimer et al., 2012; Arrieta et al., 2015; Bell-Dereske et al., 2017). However, studies considering the simultaneous interaction between plants and both endophytes and soil microbes are limited. At present, the reported research has focused on the interaction between AMF and *Epichloë* endophytes (Liu et al., 2017; Zhou et al., 2018). Some studies have found that the effects of endophytes on plant growth and competition are regulated by soil microbes (Liu et al., 2020a; Qu et al., 2023). Here, we selected the natural grass *Achnatherum sibiricum* as the experimental material. *A. sibiricum* is a perennial grass and subdominant species widely distributed in *Leymus chinensis* and *Stipa grandis* communities. In this study, we used endophyte-infected (E+) and endophyte-free (E-) *A. sibiricum* as plant materials to construct artificial communities with different diversity levels. Specifically, we aimed to test (1) whether endophyte infection has an effect on host community productivity and diversity indices (evenness), (2) whether endophyte infection affects the relationship between community species diversity and productivity (over-yielding), and (3) how endophyte infection and soil microorganisms contribute to diversity effects.

## 2 Materials and methods

### 2.1 Sampling

The plant seeds and cultivated soil were sampled at a field site of the Inner Mongolia Grassland Ecosystem Research Station of the Chinese Academy of Sciences (116°42′E, 43°38′N), where the average annual temperature is approximately 0.3°C and the average annual rainfall is approximately 355 mm. The soil total C content was 7.3 mg/g, and the soil total N content was 1.05 mg/g. Plant seeds included *Achnatherum sibiricum* (C3 grass), *Stipa grandis* (C3 grass), *Artemisia frigida* (forb) and *Cleistogenes squarrosa* (C4 grass). *A. sibiricum* is a caespitose perennial grass native to the Inner Mongolia steppe of China. This grass is normally a subordinate species in grassland but can sometimes become dominant (Ma et al., 1985). *A. sibiricum* is usually colonized by *Epichloë* endophytes with high infection rates (86–100%) in natural habitats (Wei et al., 2006). *A. sibiricum* can harbor two different *Epichloë* species, *Epichloë sibirica* and *E. gansuensis*, in different individuals of the same geographic population (Zhang et al., 2009); however, earlier studies showed that the growth and metabolism of *A. sibiricum* is significantly influenced by infection by *Epichloë* endophytes but not by the species identity of the *Epichloë* endophytes (Zhou et al., 2019; Deng et al., 2022). Therefore, in the present study, we only considered whether the plants were infected with *Epichloë* endophytes and not the species of *Epichloë* endophytes. The aniline blue staining method was used to analyze the endophyte infection frequency of seeds (Latch et al., 1984). To obtain E-seeds, we heated a subset of randomly chosen seeds for 30 days at 60°C in a convection drying oven as per Ren et al. (2011) and Li et al. (2013). Previous studies have shown that high-temperature processing inactivates *Epichloë* endophytes in seeds of *A. sibiricum* and has no significant effect on the seed germination rate, germination potential or germination index (Li et al., 2010). Similar methods have also been used in other plants, such as grove bluegrass (*Poa alsodes*) (Kannadan and Rudgers, 2008). Seeds of E+ and E- *A. sibiricum* and seeds of *S. grandis*, *A. frigida* and *C. squarrosa* were surface-sterilized in a 0.5% sodium hypochlorite solution for 10 min and washed repeatedly with sterile water until no chlorine smell was detectable. Sterilized seeds were sown into sterilized vermiculite to sprout according to their germination time. After 4 weeks of growth, the presence of *Epichloë* endophytes in *A. sibiricum* was verified from randomly selected individuals in each pot by examining the leaf sheaths for the presence of fungal hyphae after staining with lactophenol aniline blue. 50 E+ plants and 50 E- plants were examined under a microscope. The percentage of infection was 100% for the E+ plants and 0% for the E- plants. After confirming treatments in a random subselection of germinated seedlings, plants were transplated into plastic pots. Each microcosm (pot with a diameter of 29.6 cm and height of 19.7 cm) was filled with 5 kg of homogenized soil. The soil was previously passed through a 2 mm sieve in order to remove coarse fragments.

### 2.2 Experimental design

A three-factor randomized block design was used. The first factor was the endophyte infection status of *A. sibiricum*, including E+ and E-. The second factor was the presence of soil microbes, including live soil and sterilized soil. In the sterilized soil treatment, each pot was filled with 5 kg autoclaved sterilized soil (121°C for 1 h, 2 times at 24 h intervals) (McCarthy-Neumann and Kobe, 2010). The ratio of sterilized soil to live soil in the live soil treatment was 3:1. The third factor was community diversity level. Three diversity levels were set up: one-species monoculture, 2-species mixture and 4-species mixture. The one-species monoculture treatments included *A. sibiricum* (E+, E-), *S. grandis*, *A. frigida* and *C. squarrosa.* The 2-species mixture treatments included *A. sibiricum* mixed with *S. grandis*, *A. frigida* or *C. squarrosa*. The 4-species mixture treatments were a mix of four species, *A. sibiricum*, *S. grandis*, *A. frigida* and *C. squarrosa.* Twelve individuals were equally spaced and randomly assigned in the one-species monoculture treatments. In the mixed treatments, the number of individuals of different species was set according to their different aboveground coverages. When *A. sibiricum* was mixed with *S. grandis*, 6 + 6 individuals were planted per pot. When *A. sibiricum* was mixed with *A. frigida* or *C. squarrosa*, 8 *A. sibiricum* individuals + 4 A*. frigida* or *C. squarrosa* individuals were planted per pot. For the 4-species mixture treatments, 4 A*. sibiricum* individuals + 4 *S. grandis* individuals + 2 A*. frigida* individuals + 2 C*. squarrosa* individuals were planted per pot. Each combination was replicated 5 times, for a total of 130 pots. The experiment was conducted in a greenhouse at the College of Life Sciences, Nankai University. The microcosms were randomly placed under a 5-meter-height rain-proof shed to avoid natural precipitation, and their positions were changed every two weeks to avoid any position effects. The experiment began in October 2020. During the experiment, each pot was watered two-three times a week, and to ensure that the soil remained moist, the pots were watered weekly to field capacity with at least 1 L of tap water. Nutrients were supplied by the addition of Hoagland nutrient solution once per week to ensure the normal growth of plants. The experiment lasted 6 months. At the end of the experiment, aboveground shoots and belowground roots were harvested individually. The aboveground and belowground biomasses were weighed after drying at 80°C to constant weight. The community-level aboveground and belowground biomass was calculated as the sum of the biomass of each individual in the microcosm.

### 2.3 Indicator calculation

In this experiment, Shannon-Wiener diversity index (H’) for plant communities was calculated. For the pots that contained the same number of species (and therefore have the same value for species richness), we used biomass to calculate the Shannon-Wiener diversity index, reflecting changes in community evenness (Collins and Foster, 2009; Yang et al., 2015; Wu et al., 2019).


$$H'=-\sum_{i=1}^{N}(\frac{b_{i}}{\sum_{i=1}^{N}b_{i}}\times 100)\times \text{ln}\frac{b_{i}}{\sum_{i=1}^{N}b_{i}}\times 100$$

*N* is the total number of species in the community, and *b_i_* is the biomass of the i-th species.

Species abundance is the percentage of biomass of species i to the total biomass (Yang et al., 2015).

The over-yielding or net diversity effect, selection and complementarity effect on aboveground biomass and belowground biomass of each pot planted in the mixture were calculated as follows (Loreau and Hector, 2001):


$$\Delta Y=Y_{O}-Y_{E}=\sum_{i=1}^{N}RY_{Oi}M_{i}-\sum_{i=1}^{N}RY_{Ei}M_{i}=\sum_{i=1}^{N}\Delta RY_{i}M_{i}=N\bar{\Delta RY}\bar{M}+Ncov\left(\Delta RY,M\right)$$

$$Y_{E}=\sum_{i=1}^{N}\left({\text{Biomass}}_{\text{in monoculture}}\times \text{the relative richness in mixture}\right)$$

ΔY , $N\bar{\Delta RY}\bar{M}$ and Ncov(ΔRY,M) are measures of the net diversity effect, complementarity effect and selection effect, respectively. ΔY represents the deviation of the observed biomass yield (*Y_O_*) from the total expected biomass yield (*Y_E_*) in the mixture. *Y_E_* is calculated by referring to Yang et al. (2021). If ΔY is greater or lower than zero, it indicates that the community produces over-yielding or under-yielding effect. *M_i_* is the average biomass yield of species i in monoculture; *RY_oi_* = *Y_oi_*/*M_i_* is the observed relative yield of species i in the mixture; *RY_Ei_* is the expected relative yield of species i in the mixture, which is equivalent to the planted proportion; *ΔRY_i_* = *RY_Oi_* - *RY_Ei_* is the deviation from the expected relative yield of species i in the mixture; *N* is the number of species in the mixture.

### 2.4 Statistical analysis

Two-way analysis of variance (ANOVA) was used to analyze the effects of endophyte infection (E) and soil microbes (Soil) on the community biomass, Shannon–Wiener diversity index and species abundance. We used an independent samples t test to analyze the effects of species diversity (SD) and E on the over-yielding effects of total biomass and belowground biomass, and the differences in the net diversity effect, complementary effect and selection effect between the E+ and E- *A. sibiricum* in different diversity treatments. Then one sample t-test was performed to test the differences between the values of these effects and zero. Data that did not satisfy the conditions of a normal distribution and homogeneity of variance were ln-transformed prior to analysis. Analyses were performed in SPSS 26.0 (IBM, USA). To determine the contribution of endophyte infection and soil microorganisms to diversity effects, permutational multivariate analysis of variance (PERMANOVA) was performed on the variance explanation of the belowground net diversity effect with the fixed factors being infection by *Epichloë* endophytes and presence of soil microbes; the number of permutations was set to 999, and the analysis was based on Euclidean distances and calculated using the vegan package in R4.2.0.

## 3 Results

### 3.1 Effects of *Epichloë* endophyte infection on community biomass and diversity

**Figure 1:**
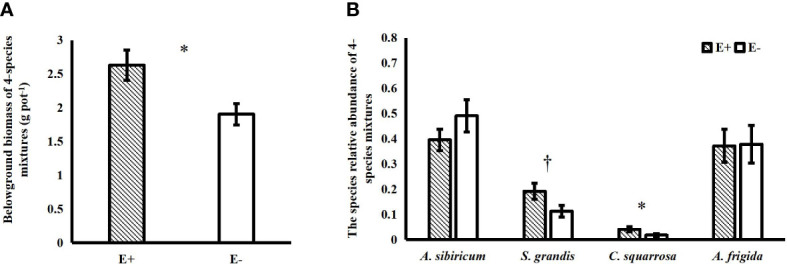
Effects of endophyte infection (E+: endophyte-infected; E-: endophyte-free) on the belowground biomass of the 4-species mixtures **(A)**; effects of endophyte infection on the abundance of each species (*Achnatherum sibiricum*, *Stipa grandis*, *Artemisia frigida* and *Cleistogenes squarrosa*) of the 4-species mixtures **(B)** (values are means ± standard errors, ∗ and † indicate differences between groups at *P*< 0.05 and *P*< 0.1, respectively).

**Figure 2:**
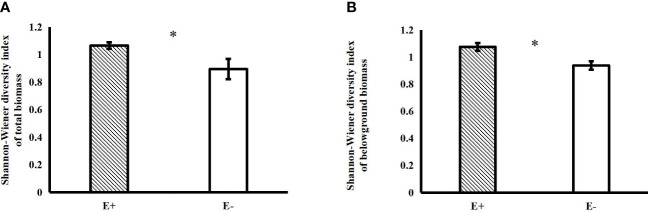
Effects of endophyte infection on the total **(A)** and belowground **(B)** Shannon-Wiener diversity index in the 4-species mixtures (E+: endophyte-infected; E-: endophyte-free, values are the mean ± standard error, ^✱^ indicates differences between groups at *P*< 0.05).

*Epichloë* endophyte infection significantly increased the belowground biomass of 4-species mixtures (**Figure 1A**). In the 4-species mixtures, *Epichloë* endophyte infection significantly increased the abundance of *C. squarrosa*, marginally significantly increased the abundance of *S. grandis* (*T*=2.021, *P*=0.058), and significantly increased the Shannon–Wiener diversity index of the 4-species mixtures (**Table 1**; **Figures 1B**, **2**).

**Table 1:** Analysis of variance (ANOVA) of endophyte infection (E), soil microbes (Soil) and their interactions (E×Soil) on the community biomass, Shannon‒Wiener diversity index and the abundance of Achnatherum sibiricum, Stipa grandis, Artemisia frigida and Cleistogenes squarrosa in the 2- and 4-species mixtures.

		2-species mixtures																			
		Biomass						Shannon‒Wiener diversity index						Abundance							
	df	aboveground		belowground		total		*A. Sibiricum* vs *S. grandis*		*A. Sibiricum* vs *C. squarrosa*		*A. Sibiricum* vs *A. frigida*		*A. sibiricum*		*S. grandis*		*C. squarrosa*		*A. frigida*	
		*F*	*P*	*F*	*P*	*F*	*P*	*F*	*P*	*F*	*P*	*F*	*P*	*F*	*P*	*F*	*P*	*F*	*P*	*F*	*P*
E	1	0.431	0.514	4.598	**0.037**	0.898	0.348	0.004	0.953	0.374	0.551	0.206	0.657	0.124	0.726	0.001	0.971	0.386	0.545	0.009	0.928
Soil	1	0.183	0.671	0.069	0.794	0.117	0.734	0.346	0.564	1.780	0.205	0.045	0.834	1.350	0.251	0.790	0.387	2.647	0.128	0.254	0.622
E×Soil	1	0.036	0.850	0.716	0.401	0.155	0.695	4.771	**0.044**	1.126	0.308	0.284	0.602	0.724	0.399	0.303	0.589	1.361	0.264	2.207	0.160
4-species mixtures																					
		Biomass						Shannon‒Wiener diversity index						Abundance							
	df	aboveground		belowground		total		aboveground		belowground		total		*A. sibiricum*		*S. grandis*		*C. squarrosa*		*A. frigida*	
		*F*	*P*	*F*	*P*	*F*	*P*	*F*	*P*	*F*	*P*	*F*	*P*	*F*	*P*	*F*	*P*	*F*	*P*	*F*	*P*
E	1	1.194	0.291	6.435	**0.022**	1.979	0.179	4.131	0.059	10.229	**0.006**	4.787	**0.044**	1.967	0.180	3.808	0.069	5.962	**0.027**	0.005	0.946
Soil	1	0.238	0.632	0.000	0.988	0.204	0.657	0.081	0.779	0.146	0.708	0.032	0.859	0.539	0.474	0.661	0.428	2.933	0.106	0.000	0.994
E×Soil	1	1.299	0.271	0.441	0.516	1.332	0.265	0.089	0.770	2.415	0.140	0.362	0.556	6.170	**0.024**	0.128	0.725	0.408	0.532	2.787	0.114

Bold in the table represents significant differences (*P*< 0.05).

### 3.2 Effects of *Epichloë* endophyte infection and soil microbes on community over-yielding

**Figure 3:**
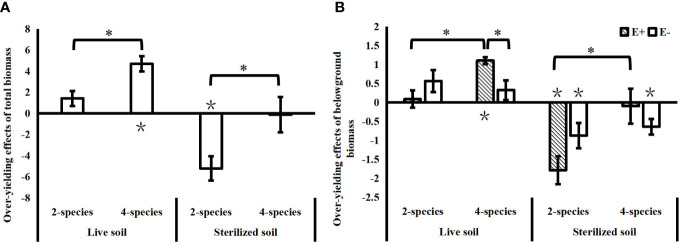
Effects of species diversity (SD), soil microbes and endophyte infection (E+: endophyte-infected; E-: endophyte-free) on the over-yielding effects of total community biomass **(A)** and belowground biomass **(B)** (values are the mean ± standard error; ∗ indicates differences between SD groups and endophyte infection groups at *P*< 0.05; ∗ indicates significant differences between over-yielding effect values and 0).

In the live soil, the community over-yielding effects increased significantly with species diversity, while in the sterilized soil, the community under-yielding effects decreased significantly with increasing species diversity (**Figure 3A**). Soil microbes in the live soil increased the community biomass compared to the sterilized soil, as evidenced by a significant reduction in the under-yielding effects of soil microorganisms in 2-species mixtures (*T*=4.922, *P*<0.001) and a significant increase in community over-yielding effects in 4-species mixtures (*T*=2.649, *P*=0.021). *Epichloë* endophyte did not affect the total community biomass over-yielding effect, and the effects of *Epichloë* endophytes on community over-yielding occurred only belowground in the 4-species mixtures in the live soil, and *Epichloë* endophyte infection significantly increased the belowground community over-yielding effects. With increasing species diversity, endophyte-infected communities not only showed significant increases in belowground over-yielding effects in the live soil, but also significantly alleviated the belowground under-yielding effects of communities in the sterilized soil. In contrast, there was no significant change in the belowground over-yielding effects of endophyte-uninfected communities in the live soil and no significant change in belowground under-yielding effects in the sterilized soil (**Figure 3B**).

### 3.3 Effects of endophyte infection on community diversity and diversity effects

**Figure 4:**
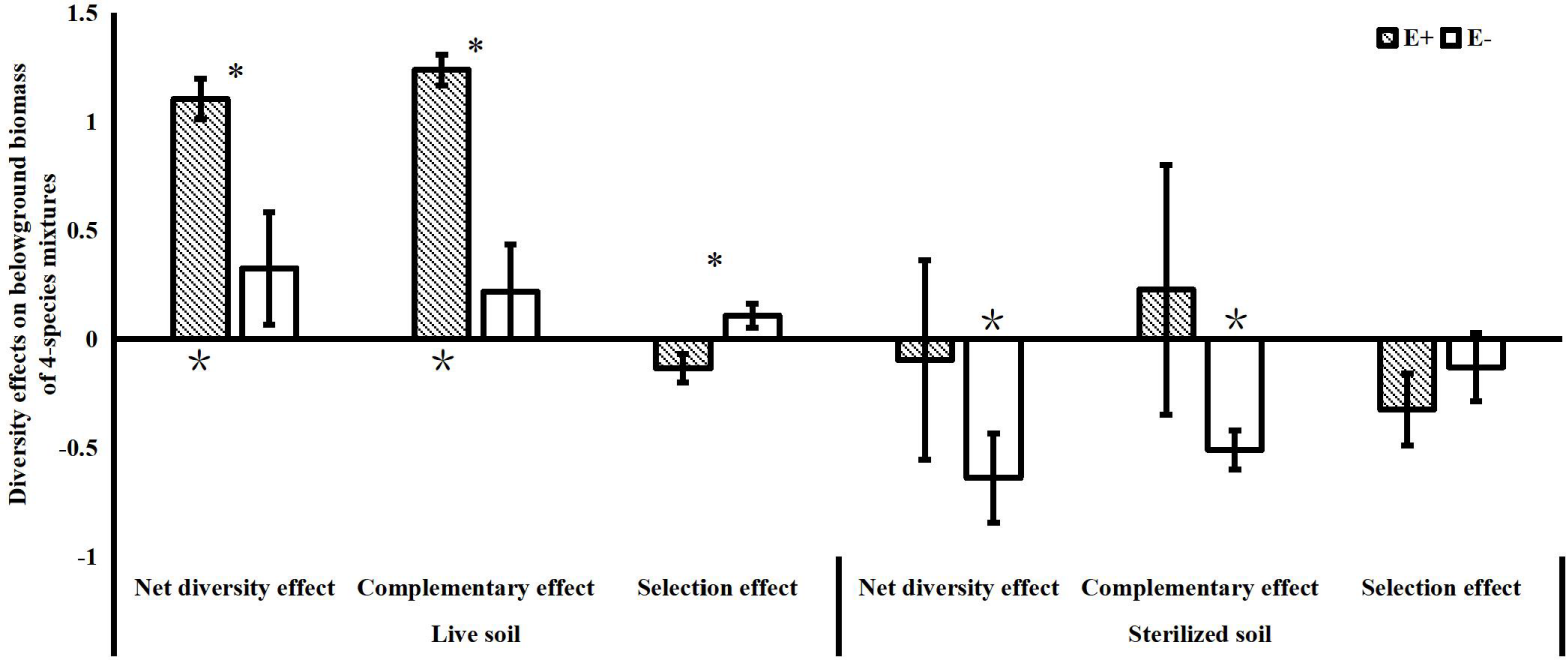
Net diversity effects, complementary effects and selection effects of belowground biomass in 4-species mixtures (E+: endophyte-infected; E-: endophyte-free, values are means ± standard errors; ∗ indicates differences between groups at *P*< 0.05; * indicates significant differences between belowground diversity effect values and 0).

The effects of endophytes on community net diversity were mainly observed belowground in the 4-species mixtures in our study. We found that endophyte infection significantly increased the complementary effects on belowground biomass of the 4-species mixtures in the live soil treatments. Although endophytes significantly reduced the selection effects on belowground biomass of the community, the values of the selection effects were not significantly different from 0 with or without endophyte infection. In the sterilized soil, endophyte infection also reduced the negative values of the complementary effects on belowground biomass (**Figure 4**).

### 3.4 Variance contributions of endophytes and soil microbes to community diversity effects

**Figure 5:**
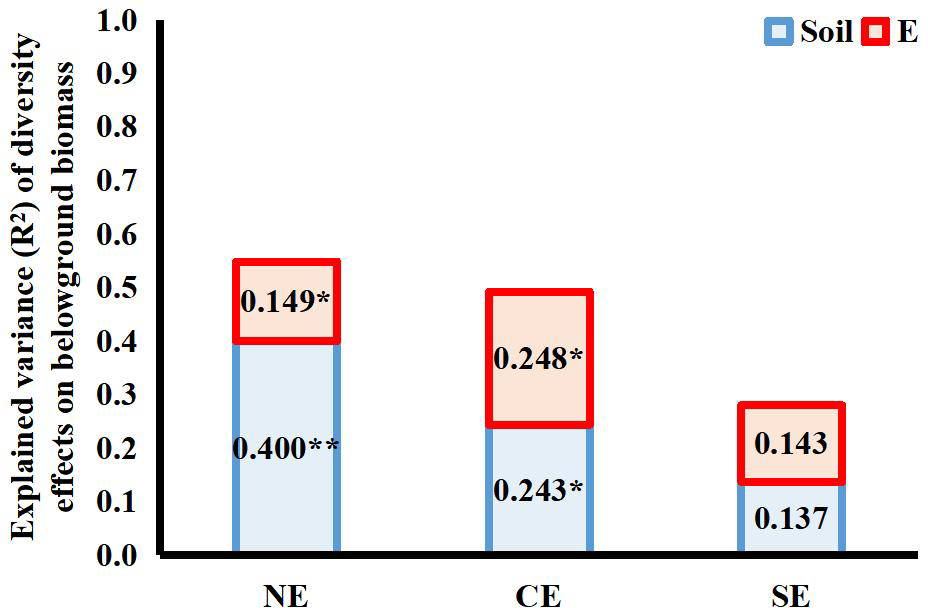
Explanation of variance (R^2^) for the effects of endophyte infection (E) and soil microbes (Soil) on net diversity effects (NE), complementary effects (CE) and selection effects (SE) on belowground biomass in the 4-species mixtures (numbers in the figure represent R^2^ values; * and ** indicate differences between groups at *P*< 0.05 and *P*< 0.01, respectively.

Both endophytes and soil microbes contributed significantly to the variance in the net diversity effects on belowground biomass of the 4-species mixtures, but the interaction between the two explained very little of the total variance (net diversity effect: R^2^ = 0.005; complementary effect: R^2 ^= 0.006; selection effect: R^2 ^= 0.002). Both endophytes and soil microbes had a significant effect on the complementary effect on belowground biomass, and both had similar contributions. Neither endophytes nor soil microbes contributed significantly to the selection effect on belowground biomass in terms of variance, as endophyte infection reduced the selection effect but increased the complementary effect, and the different directions of the effects resulted in a smaller variance contribution value of endophyte infection to the net effect on belowground biomass than to the complementary effect on belowground biomass (**Figure 5**).

## 4 Discussion

### 4.1 Effects of endophytes on community diversity and productivity

Endophyte infection can affect host plant growth and interspecific competition and thus may also affect the diversity and productivity of the host community, but the number of related studies thus far is still low. In the present study, infection with endophytes significantly increased the community diversity (evenness) and belowground biomass of 4-species mixtures but had no significant effect on either aboveground or total community biomass. This is similar to the findings of Afkhami and Strauss (2016), who found that infection with endophytes resulted in an increase in community diversity, mainly resulting in a significant increase in community evenness, but no significant change in community aboveground biomass. It has also been found that endophyte infection does not affect the aboveground biomass of the community but reduces community species diversity (Clay and Holah, 1999). The reason for the different results may be related to the host plant selected for the study; Afkhami and Strauss (2016) used the natural grass *B. laevipes*, and the *A. sibiricum* selected for our study is also a natural grass, while Clay and Holah (1999) used the cultivated grass *Festuca arundinacea*. The relationship between the natural grass–endophyte symbionts and other species in the community was developed through a long successional process under biotic and abiotic selection pressures, so the effects of the natural grass–endophyte symbionts on other species in the community may differ from the effects of the newly introduced cultivated grass–endophyte symbionts. In a study by Afkhami and Strauss (2016), endophytes increased community diversity by promoting the growth of nondominant species. In the present study, endophytes also increased community diversity (evenness) by increasing the abundance of *C. squarrosa* and *S. grandis*, which are less abundant species, suggesting that endophyte infection facilitates the maintenance of host community diversity. Unlike the abovementioned studies, the present study measured the effects of endophytes on both aboveground and belowground biomass of the communities separately and found that endophyte infection significantly increased the belowground biomass of the 4-species mixtures.

### 4.2 Effects of endophytes on community diversity effects

In this study, we found that endophyte infection in 4-species mixtures in live soil significantly increased the diversity effects of community belowground biomass, mainly by significantly increasing the complementary effects on belowground biomass. As far as we know, we are the first to study how *Epichloë* endophyte infection affect the complementary and selection effects on community biomass. The significant effects of endophyte infection on complementary effects of community belowground biomass may be reached in following ways. Firstly, endophyte infection can increase the amount of available resources such as organic carbon and nitrogen in the soil of both cultivated grasses (Franzluebbers et al., 1999; Iqbal et al., 2012; Chen et al., 2021) and natural grasses (Zhou et al., 2014; Liu et al., 2020b; Hou et al., 2020). Endophyte infection leading to increased soil availability may be associated with the ability of endophyte infection to increase host root biomass (Franzluebbers, 2006) because more vigorous root growth can enhance rhizosphere deposition of organic carbon and carbohydrates (Van Hecke et al., 2005), and endophytes can also affect soil enzymes and thus soil fertility (Hou et al., 2020). Secondly, the significant effects of endophyte infection may also influence complementary effects by affecting the rate of AMF colonization, which can promote plant nutrient uptake and expand the community resource niche. AMF form symbiotic associations with 80% of all terrestrial plant species to enhance their nutrient acquisition capacity and greatly improve the efficiency of soil nitrogen and phosphorus acquisition (Facelli et al., 2010; Turrini et al., 2018). Recent studies suggest that endophytes can not only affect the AMF colonization rate of the host plant, but also affect AMF species diversity in the soil and thus the colonization rate of neighboring nonhost plants (Novas et al., 2005; Novas et al., 2009; Liu et al., 2020b). For instance, Omacini et al. (2006) found that although endophyte infection suppressed AMF colonization rates in host ryegrass roots, AMF colonization rates in the roots of uninfected neighbors increased. In the present study, we found that endophyte infection increased the abundance of *C. squarrosa* and *S. grandis*, but did not affect the abundance of *A. frigida*. A possible explanation is that *A. frigida* is a forb while the other species in the 4-species mixture are grasses. The ecological niche differentiation might make *A. frigida* compete less with other species for resources, but there might be more intense interspecific competitive effects among the roots of *A. sibiricum*, *C. squarrosa*, and *S. grandis*. Endophyte infection may alleviate the competitive effects among *A. sibiricum*, *C. squarrosa*, and *S. grandis*, increase the complementary of the belowground community, and thus increase the belowground biomass.

### 4.3 Contribution of endophytes and soil microorganisms to the community diversity effect

In this study, soil microorganisms significantly attenuated the community under-yielding effects in the 2-species mixtures and significantly increased the community over-yielding effects in the 4-species mixtures, indicating that soil microorganisms can increase the community net diversity effects. Our study is similar to previous studies that reported that soil microorganisms can drive a positive plant diversity–productivity relationship and promote community over-yielding (Schnitzer et al., 2011; Wang et al., 2019; Yang et al., 2022) Soil microorganisms can influence diversity effects of community biomass in several ways. Schnitzer et al. (2011) concluded that the positive relationship between diversity and productivity is mainly due to the dilution of soil pathogens in high-diversity communities and the decrease in host-specific diseases, resulting in increased productivity. Wang et al. (2019) found that the positive diversity effects caused by soil microorganisms were mainly derived from selection effects, with the effects of soil microorganisms on the dominant species *Andropogon gerardii* leading to community over-yielding. In this study, the significant contributions of aboveground endophytes and soil microorganisms to the diversity effects of community belowground biomass in the 4-species communities were derived from the significant effects of both on the complementary effects. Although the contribution of endophyte infection to the complementary effects on belowground biomass was similar to that of soil microorganisms, endophyte infection in live soil significantly reduced the selection effects on belowground biomass of the communities, which may have resulted in a lower contribution of endophyte infection to the net diversity effects than that of soil microorganisms. Although the selection effects on belowground biomass were weaker in the live soil, endophyte infection still significantly reduced the selection effects, exhibiting suppressive effects on dominant highly productive species and leading to an increase in community evenness. The effects of endophytes and soil microbes on diversity effects of community biomass were independent in this study, which is inconsistent with results showing that endophytes can influence soil microbial communities (Buyer et al., 2011; Bell-Dereske et al., 2017). A possible reason is that the planting time of this experiment was relatively short, and the significant effects of endophytes on soil microorganisms may gradually become apparent with the extension of the experimental time. The significant effects of endophytes on the diversity effects of community belowground biomass suggest that endophyte infection is beneficial to the growth of multiple species in the habitat, which explains the stable co-existence of various plants, such as *S. grandis*, *A. frigida* and *C. squarrosa*, with a high endophyte infection rate of *A. sibiricum* in the stable community of Inner Mongolia.

## Data availability statement

The raw data supporting the conclusions of this article will be made available by the authors, without undue reservation.

## Author contributions

AR and YQ conceived and designed the experiments. YQ, TQ, JZ, YD and XY performed the experiments. YQ, XW, NZ and AR analyzed the data. YQ, YG and AR wrote the manuscript; others provided editorial advice. All authors contributed to the article and approved the submitted version.
